# Cloning of a Novel Protein Interacting with BRS-3 and Its Effects in Wound Repair of Bronchial Epithelial Cells

**DOI:** 10.1371/journal.pone.0023072

**Published:** 2011-08-09

**Authors:** Hui Jun Liu, Yu Rong Tan, Meng Lan Li, Chi Liu, Yang Xiang, Xiao Qun Qin

**Affiliations:** Department of Physiology, Xiangya School of Medicine, Central South University, Changsha, China; National Institutes of Health, United States of America

## Abstract

Bombesin receptor subtype 3 (BRS-3), the orphan bombesin receptor, may play a role in the regulation of stress responses in lung and airway epithelia. Bombesin receptor activated protein (BRAP )is a novel protein we found in our previous study which interacts with BRS-3. This study was designed to observe the subcellular location and wound repair function of BRAP in human bronchial epithelial cells (HBECs). BRAP ORF was amplified by RT-PCR and ligated to pEGFP-C1 vector, and then the recombinant plasmid pEGFP-C1-BRAP was transfected into Hela cells. The location of BRAP protein was observed by laser confocal microscope, and the expression of it was analyzed by Western-blot. At the same time,we built the recombinant plasmid pcDNA3.1(+)-BRAP, transfected it into HBECs and observed its impact on cell cycle and wound repair of HBECs. The results showed that BRAP locates in membrane and cytoplasm and increases significantly in transfected cells. Flow cytometry results demonstrated that the recombinant plasmid increases S phase plus G2 phase of cell cycle by 25%. Microscopic video analysis system showed that the repair index of wounded HBECs increases by 20% through stable expression of BRAP. The present study demonstrated that BRAP locates in the membrane and cytoplasm, suggesting that this protein is a cytoplasm protein, which promotes cell cycle and wound repair of HBECs.

## Introduction

There is accumulating evidence that BLPs and their receptors play an important role in pathological conditions of the lung, including chronic inflammatory lung disease, lung cancer, bronchopulmonary dysplasia and acute lung injury. Bombesin receptor subtype-3 (BRS-3, BB3) is a GPCR that has been resistant to deorphanization. First discovered in 1992 through homology screening approaches, BRS-3 was assigned to the bombesin receptor family for its high sequence similarity to two mammalian bombesin receptors: 47% to Neuromedin B receptor (NMB-R, BB1) and 51% to Gastrin-releasing peptide receptor (GRP-R, BB2) [1], [2].

Unlike GRP and NMB receptors, which have a widespread distribution in the central nervous system and peripheral tissues, the distribution of BRS-3 in the majority of normal tissues is extremely low, whereas its level in the developing lung and certain lung carcinomas is high [3]–[5]. There is very little known about the biological function of BRS-3 activation. This is in great part because the natural ligand of BRS-3 has not yet been identified. However, results from our previous experiments demonstrated that the expression of BRS-3 mRNA was significantly up-regulated in an ozone-stressed airway hyperresponsiveness animal model and resulted in wound repair and Th1-type immune response [6]–[7]. Using bacterial two-hybrid technology, we observed that BRS-3 can interact with a variety of proteins which are related with cell growth, differentiation, anti-apoptotic, cytoskeleton assembly, tyrosine kinase activation and so on. We also found that BRS-3 interacts with a new protein named as bombesin receptor activated protein( BRAP) in present study.

To further study the structure and function of BRAP, its subcellular localization and effects on cell proliferation and wound repair were studied, which will provide theoretical basis for the interaction of BRAP and BRS-3 in stress response of bronchial epithelial cells.

## Materials and Methods

### Cell lines and culture

An immortalized human bronchial epithelial cell line(16HBE14o-) and Hela cell line were maintained in a mixture medium of DMEM∶F12(1∶1) supplemented with 100 µg/ml penicillin, 100 µg/ml streptomycin and 10% newborn bovine serum. Cells were incubated at 37°C in a humidified incubator with 5% CO_2_.

### Cloning of BRAP from HBECs

Total RNA was isolated from HBECs using TRIzol reagent (Invitrogen, USA) according to the manufacturer's protocol. Superscript RNase ^H-^ Reverse Transcriptase (Invitrogen) and an oligo (dT) primer were used to synthesize the first strand cDNA. PCR reaction (5′-GCCC aga tct GAT TTT ATA TTG GAA GAC-3′ forward and 5′-GCCA ctg cag CAG TTC TGA GAA AGC GC-3′ reverse) was performed as follows: 94°C for 5 min, then 30 cycles of 94°C for 30 s, 47°C for 30 s,72°C for 45 s, and then a final extension at 72°C for 10 min. Amplification of beta-actin (5′-AGCGCGAAATCGTGCGTG-3′ forward and 5′-CAGGGTACATGGTGGTGCC-3′reverse) was used as a control for sample loading and normalization. The primers used were listed in Table 1. RT-PCR products were ligated into a pGEM-T-easy vector. Subsequently, the plasmid was transformed into E.coli strain DH5. Bacteria were cultured in 800 µL of LB medium for 45 min at 37°C and 225r/min. After incubation, bacteria were plated onto agar plates containing ampicillin(100 µg/L), X-gal(20 µg/cm^2^)and IPTG(12 µg/cm^2^) and incubated overnight at 37°C.White colonies were selected and identified by sequencing.

**Table 1:** Primers used in this study.

Primers name	primer sequence(5′-3′)	purpose
FP1	GCCC aga tct GAT TTT ATA TTG GAA GAC	RT-PCR
RP1	GCCA ctg cag CAG TTC TGA GAA AGC GC	RT-PCR
FP2	GCCC aga tct GAT TTT ATA TTG GAA GAC	pEGFP-C1-BRAP
RP2	GCCA ctg cag CAG TTC TGA GAA AGC GC	pEGFP-C1-BRAP
FP3	GCCC gga tcc GAT TTT ATA TTG GAA GAC	pcDNA3.1(+)-BRAP
RP3	GCCA ctc gag CAG TTC TGA GAA AGC GC	pcDNA3.1(+)-BRAP
FP—actin	AGCGCGAAATCGTGCGTG	beta-actin
RP—actin	CAGGGTACATGGTGGTGCC	beta-actin

### Construction of BRAP expression vectors

pEGFP-C1 and pcDNA3.1 (+) vectors were purchased from clontech (USA). The transcript BRAP was digested by BglII/PstI and inserted into pEGFP-C1 and pcDNA3.1(+) plasmids, respectively. Both constructs were confirmed by sequencing and digest.

### Cell transfection and fluorescence imaging

The expression vectors were transfected into Hela and HBECs using LipofectamineTM 2000 reagent (Invitrogen) according to the manufacturer's protocol. Empty pEGFP-C1 and pcDNA3.1 (+) plasmids were transfected respectively as control. After pEGFP-C1-BRAP plasmid was transfected into Hela 48 hours later, GFP expression was recorded using confocal laser scanning microscopy. pcDNA3.1(+)-BRAP plasmid was transfected into HBECs. Positive cell clones were obtained by antibiotic selection for 2 weeks with G418 (Gibco,Grand Island,NY) at a concentration of 600 ug/ml. The stable transfectants used in this study were stained with propidium iodide and analyzed by flow cytometry and monolayer wound repair assay.

### Protein extraction and Western blotting analysis

The level of protein expression was evaluated by Western blotting analysis using specific antibodies. Hela cells transfected with pcDNA3.1 (+) and pcDNA3.1 (+)-BRAP were collected from the plates. The samples were lysed in ice-cold cell lyses buffer (1% Triton X-100, 150 mM NaCl, 10 mM Tris-HCl (pH 7.4), 2 mM sodium orthovanadate, 10 µg/ml leupeptin, 50 mM NaF, 5 mM EDTA, 1 mM EGTA, and 1 mM PMSF) by stirring for 1 h at 4°C. The lysates were obtained after a centrifugation at 13,000×g for 15 min. An equal volume of Laemmle sample buffer was added to each cell lysate. Samples were boiled for 10 min, and then equal amounts of protein were separated by 7.5% SDS-PAGE before being transferred to nitrocellulose membrane. The membrane were blocked with 3% BSA in PBS for 2 h and then incubated with antibodies and appropriate horseradish peroxidase-conjugated secondary antibody. Detection was made using the enhanced chemiluminescene system.

### Measurement of cell cycle by flow cytometry

After being cultured in 6-well plates, cells were harvested at a density of 1×10^6^ Cells/mL, fixed in cold 70% ethanol and stored at −20°C overnight. The fixed cells were washed twice with phosphate-buffered Saline, stained in a propidium iodide solution (50 ug/ml) for 1 hour, and treated with a ribonuclease A solution (20 ug/ml) for 30 minutes. Flow cytometry was then using to examine cell cycle. Experiments were repeated 3 times.

### Monolayer wound repair assay

This assay was used to demonstrate the effect of BRAP overexpression on migration and wound repair of epithelial cells. We have previously published details of this method. Briefly [6], HBECs transfected with pcDNA3.1 (+)-BRAP were grown until confluent in 12-well plates with DMEM: F12 (1∶1), and a small wound was made in the confluent monolayer with a rubber stylet. The edge of wound was recognized and the remaining wound area was measured serially per 4 hours in 24 h by video microscopy (Olympus Company, Japan). A linear regression equation of the remaining wound area to time was obtained. Repair index (RI), equal to the absolute value of slope, was used to judge the repair speed of HBECs.

### Statistical analyses

The data were analyzed using unpaired Student's t-test. Values were expressed by mean±SE. P<0.05 was considered as statistically significant.

## Results

### Cloning and sequence analysis of BRAP transcript from HBECs

The nucleotide sequence of BRAP has been deposited in Gene Bank with accession number NM-152734. The original ORF of BRAP is composed of 1065 nt and putatively 354 amino acid residues(Figure S1A). n present study, the ORF of BRAP was generated by RT-PCR amplification from mRNA of HBECs (Figure S1B), subcloned into pGEM-T-easy vector, and identified by sequencing.

### Identification of amplified plasmids

After being digested by endonucleases BgI II and Pst I, the plasmid pEGFP-C1-BRAP (Figure S2A) showed 2 bands of about 5 Kb and 1 Kb, and restriction enzyme analysis of the plasmid pcDNA3.1(+)-BRAP with BamHI and XhoI yielded approximately 5 Kb empty pcDNA3.1(+) and 1 Kb BRAP (Figure S2B).

### Subcellular localization of BRAP

A GFP fusion expression construct, pEGFP-C1-BRAP, was transfected into cultured Hela cells in order to visualize the subcellular localization of BRAP. Fluorescence was measured at 48 h post-transfection using confocal microscopy. As shown in Figure S3, GFP-BRAP clearly locates in the membrane and cytoplasm.

### Expression of BRAP protein in transfected HBECs

To investigate the expression of BRAP protein, western blotting analysis of cell lysates was performed utilizing the specific rabbit anti-BRAP polyclonal antibody and revealed that pcDNA3.1(+)-BRAP transfected cells possesses much higher expression of BRAP compared with mock plasmid-transfected cells and untransfected cells (Figure S4) .

### Cell cycle measurement by flow cytometry

The ratio of S-phase in cells transfected with pcDNA3.1 (+)-BRAP was significantly higher than in cells transfected with pcDNA3.1 (+) and untransfected cells (Figure S5A). The ratio of S-phase was shown by columns (^*^p<0.05, Figure S5 B)

### Effects of BRAP overexpression on wound repair

PCDNA3.1 (+) - BRAP and PCDNA3.1 (+) transfected HBECs were cultured in 24 well plates to 90% confluence, and then changed into serum-free culture medium for 24 h. The cell layer was caused a minor mechanical wound by scratching and monitored for 24 h by the use of BI-2000 image immunohistochemical analysis system, outlining the edge of wound area (Figure S6 A). Compared with pcDNA3.1(+) transfected cells, the repair of HBECs was accelerated significantly after BRAP overexpression. (Figure S6 B).

## Discussion

BRS-3 is one of G protein-coupled receptors which are the largest family of cell surface molecules involving in signal transduction and represent about 3% of the human genome. By use of bacterial two-hybrid technology, we found a new protein interaction with BRS-3. Bioinformatics analysis showed that full-length cDNA of the new protein is 6751 bp and locates at 6p21.2 (access number: NM_152734), with ORF length 1065 bp encoding a 354 amino acid protein. Northern Blot analysis showed that BRAP is expressed in HBECs, embryonic tissues and tumor tissues.

Subcellular localization of proteins is an important aspect of protein function study. Protein synthesis in the ribosome, and then the boot to specific organelles, involved in a variety of cell life activities, such as cell cycle regulation, cell signal transduction and transcriptional regulation. In case of deviation, it will be of significant impact on the entire cell function [8], [9]. To study the subcellular localization of BRAP, we constructed a plasmid PEGFP-BRAP by fusion to green fluorescent protein (GFP). Using laser scanning confocal microscope, we observed BRAP locates in membrane and cytoplasm. Western blot results showed that the protein is highly expressed in transfected cells.

The signal transduction of BRS-3 in different cells or under different stimuli can lead to different cell responses depending on whether the intracellular signaling molecules assemblies with different upstream and downstream molecules, i.e. assemblies of different signaling molecules determine the different responses. Stress responses of HBECs, include cell proliferation and repair, expression of adhesion molecules and adhesion with inflammatory cells to transmit stress signals, intake and presenting antigen to activate lymphocytes and so on [10]–[13]. In the mechanism of stress signaling transmission in HBECs to produce airway hyperresponsiveness, BRAP may be involved in assembling with specific signal molecules.

BRAP was cloned into pcDNA3.1 (+) multiple cloning sites and the effects on stable over-expression of BRAP in HBECs on cell cycle were observed. The cell cycle consists of four distinct phases: G1 phase, S phase (synthesis), G2 phase (collectively known as interphase) and M phase (mitosis). Activation of each phase is dependent on the proper progression and completion of the previous one. Cells that have temporarily or reversibly stopped dividing are said to have entered a state of quiescence called G0 phase. Our results showed that, BRAP promotes transformation from G1 to S phase, accelerates cell into DNA synthesis phase, thereby promoting DNA synthesis and cell proliferation.

To further determine the effects of BPAP activation in AHR, we tried to observe the effects of BPAP on wound repair of BECs. Bronchial epithelial cells are the first line of defense against external stimuli, which are not only the mechanical barrier, but play an important role in the homeostasis of local microenvironment of the lungs. Injury or dysfunction of bronchial epithelial cells leading to imbalance of local micro-environments may be the initiating link of asthma and other airway diseases. It is reported that the wound repair mainly depends on migration and proliferation. In the prophase of wound repair, the precursor cells around the wound initiate the migration and dissemination. Then the proliferation takes place around16–20 h after the damage [14]–[15]. The results showed that BRAP promotes the repair and proliferation of BECs. However, whether the increase of wound repair is associated with increasing cell adhesion or losing contact inhibition between cells needs further research.

## Supporting Information

Figure S1 A: Depicted are 1065 base pairs of DNA sequence of the human. The predicted protein sequence is shown below the nucleotide sequence. B: BRAP mRNA expression in HBECs was assayed by RT-PCR. 1: represents β- actin; 2: represents BRAP PCR products; 3: represents DNA mark 2000 plus.
(TIF)

Figure S2 Recombinant plasmids were assayed by restriction enzyme analysis. A 1: Digestion the plasmid pEGFP-C1-BRAP by BgI II/Pst I; 2: DNA marker; 3: pEGFP-C1-BRAP. B 1: Digestion the plasmid pcDNA3.1-BRAP by Bam H1/Xho I; 2: DNA marker; 3: pcDNA3.1-BRAP.
(TIF)

Figure S3 Subcellular localization of BRAP protein in Hela cells. A: Normal Hela Cells; B: pEGFP-C1 transfected Hela cells; C pEGFP-C1-BRAP transfected Hela cells.
(TIF)

Figure S4 Expression of the BRAP-encoded protein in HBECs after transfection. A 1: cells transfected with pcDNA3.1(+)-BRAP plasmid; 2: cells transfected with mock plasmid(pcDNA3.1(+)) transfected; 3: parent cells. B Quantification of Western blots normalized to the level of B-actin.
(TIF)

Figure S5 The influence of BRAP overexpression on cell cycle of HBECs. A:1, Parent cells; 2, Cells transfected with pcDNA3.1 (+); 3, Cells transfected with pcDNA3.1 (+)-BRAP; B: S-phase cells expressed were shown by columns. (^*^p<0.05, n = 3).
(TIF)

Figure S6 The influence of BRAP on wound repair of HBECs. RI was used to evaluate the speed of wound repair. A: typical video micrographs of HBECs monolayer wound closure; B: Closure of monolayer wounds in HBECs after transfection with pcDNA3.1 (+)-BRAP. (^*^p<0.05 vs control and PCDNA3.1(+)).
(TIF)
