# Supplementary figures and images for: Cloning of a Novel Protein Interacting with BRS-3 and Its Effects in Wound Repair of Bronchial Epithelial Cells
Source: PLoS One. 2011 Aug 9;6(8):e23072. doi: 10.1371/journal.pone.0023072 (PMC3153462; doi:10.1371/journal.pone.0023072)

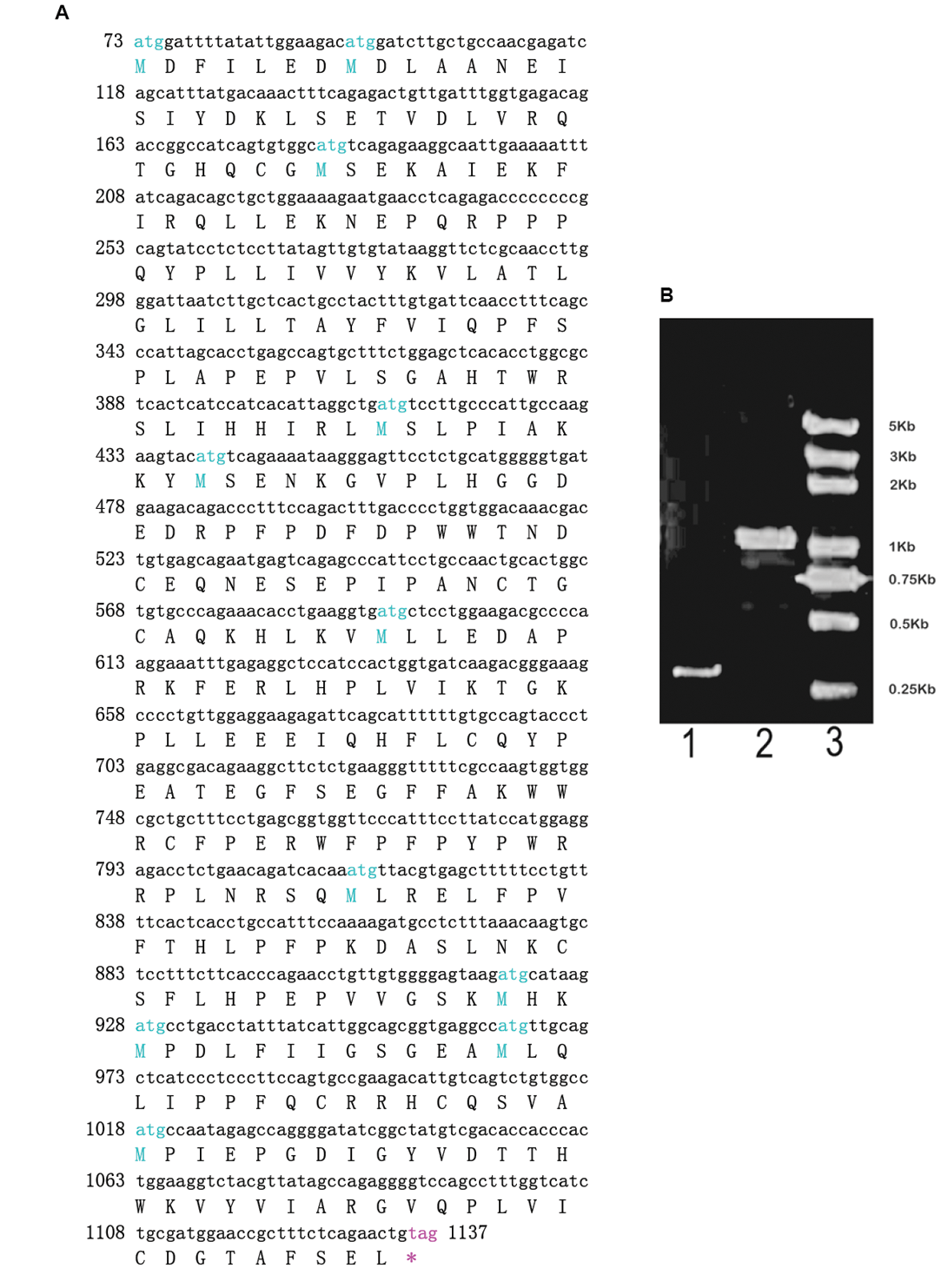

Supplement: Figure S1 — A: Depicted are 1065 base pairs of DNA sequence of the human. The predicted protein sequence is shown below the nucleotide sequence. B: BRAP mRNA expression in HBECs was assayed by RT-PCR. 1: represents β- actin; 2: represents BRAP PCR products; 3: represents DNA mark 2000 plus. (TIF) [file pone.0023072.s001.tif]

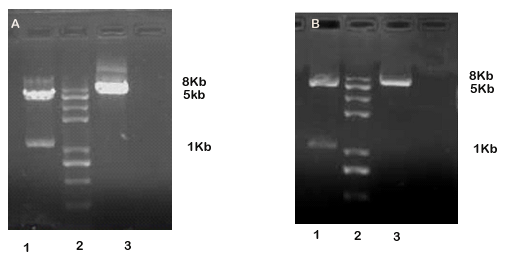

Supplement: Figure S2 — Recombinant plasmids were assayed by restriction enzyme analysis. A 1: Digestion the plasmid pEGFP-C1-BRAP by BgI II/Pst I; 2: DNA marker; 3: pEGFP-C1-BRAP. B 1: Digestion the plasmid pcDNA3.1-BRAP by Bam H1/Xho I; 2: DNA marker; 3: pcDNA3.1-BRAP. (TIF) [file pone.0023072.s002.tif]

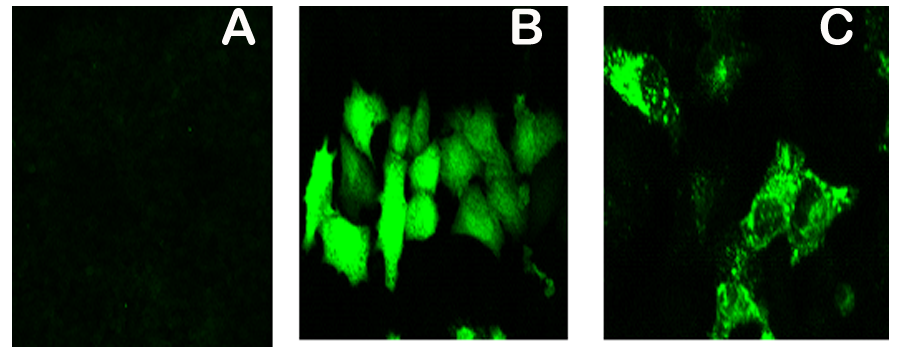

Supplement: Figure S3 — Subcellular localization of BRAP protein in Hela cells. A: Normal Hela Cells; B: pEGFP-C1 transfected Hela cells; C pEGFP-C1-BRAP transfected Hela cells. (TIF) [file pone.0023072.s003.tif]

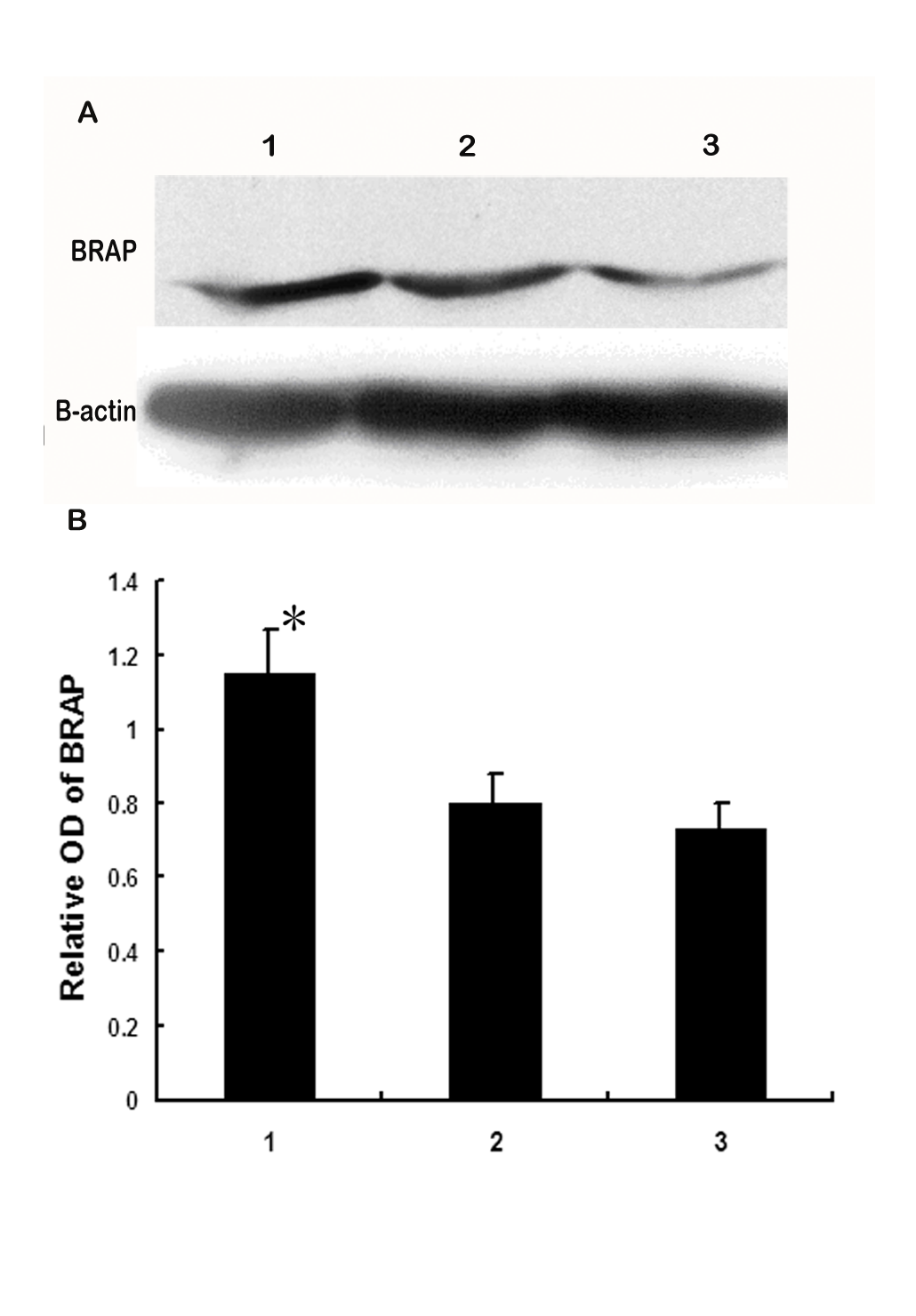

Supplement: Figure S4 — Expression of the BRAP-encoded protein in HBECs after transfection. A 1: cells transfected with pcDNA3.1(+)-BRAP plasmid; 2: cells transfected with mock plasmid(pcDNA3.1(+)) transfected; 3: parent cells. B Quantification of Western blots normalized to the level of B-actin. (TIF) [file pone.0023072.s004.tif]

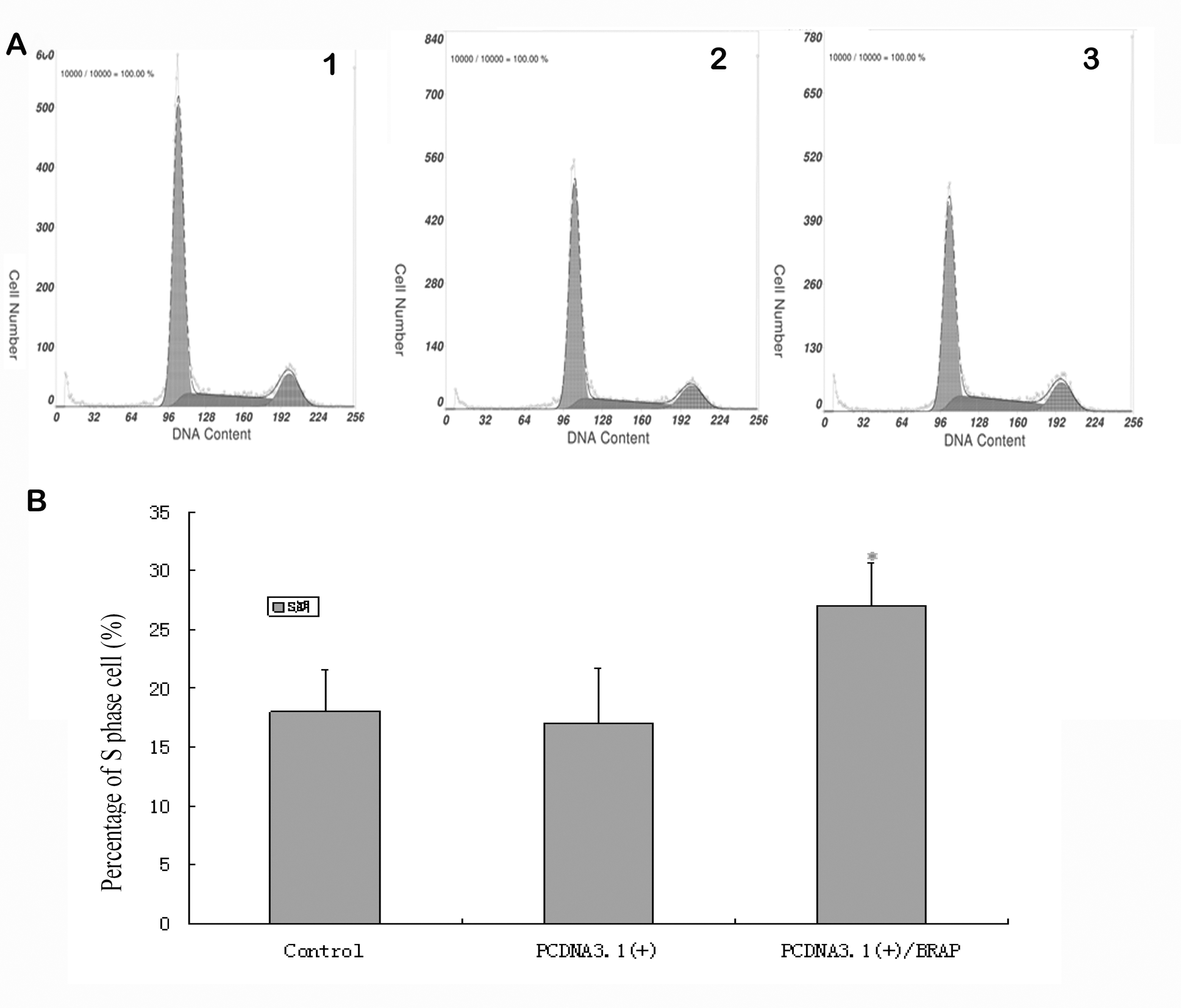

Supplement: Figure S5 — The influence of BRAP overexpression on cell cycle of HBECs. A:1, Parent cells; 2, Cells transfected with pcDNA3.1 (+); 3, Cells transfected with pcDNA3.1 (+)-BRAP; B: S-phase cells expressed were shown by columns. (*p<0.05, n = 3). (TIF) [file pone.0023072.s005.tif]

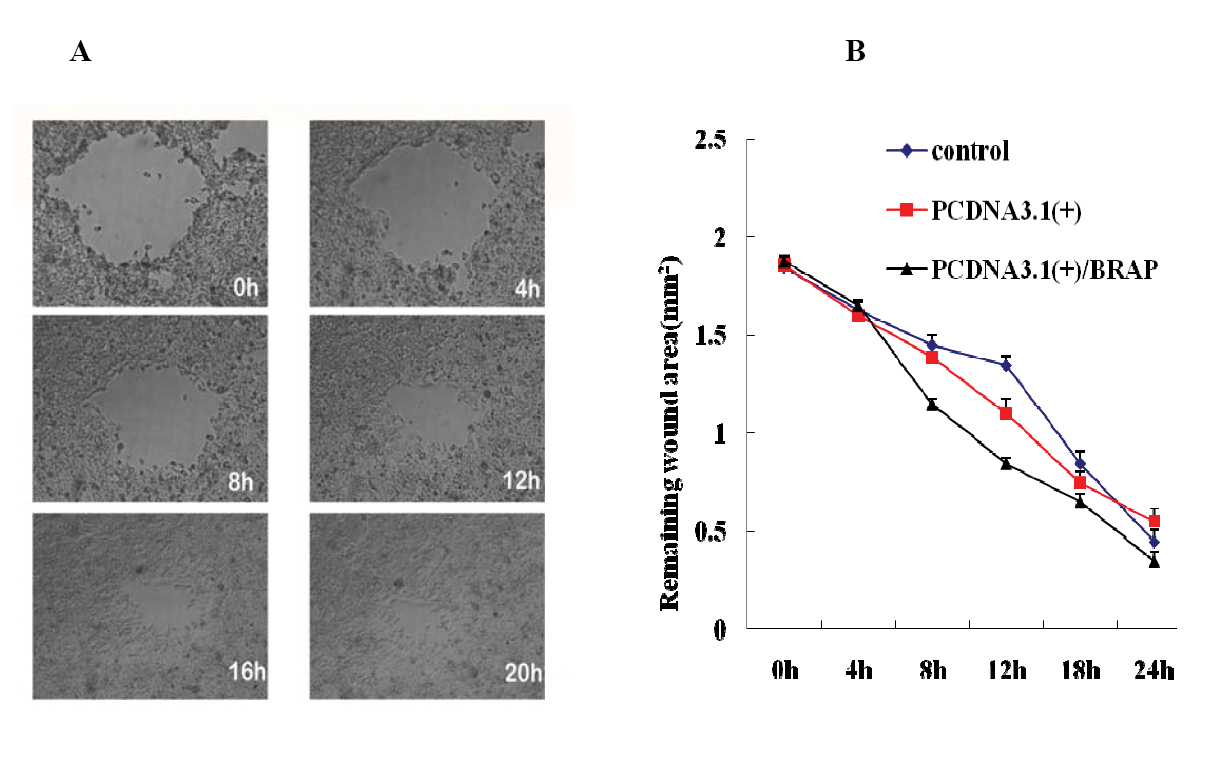

Supplement: Figure S6 — The influence of BRAP on wound repair of HBECs. RI was used to evaluate the speed of wound repair. A: typical video micrographs of HBECs monolayer wound closure; B: Closure of monolayer wounds in HBECs after transfection with pcDNA3.1 (+)-BRAP. (*p<0.05 vs control and PCDNA3.1(+)). (TIF) [file pone.0023072.s006.tif]
